# The Role of Innate Immune Responses in the Outcome of Interspecies Competition for Colonization of Mucosal Surfaces

**DOI:** 10.1371/journal.ppat.0010001

**Published:** 2005-07-22

**Authors:** Elena S Lysenko, Adam J Ratner, Aaron L Nelson, Jeffrey N Weiser

**Affiliations:** Departments of Microbiology and Pediatrics, University of Pennsylvania School of Medicine, Philadelphia, Pennsylvania, United States of America; University of California at Berkeley, United States of America

## Abstract

Since mucosal surfaces may be simultaneously colonized by multiple species, the success of an organism may be determined by its ability to compete with co-inhabitants of its niche. To explore the contribution of host factors to polymicrobial competition, a murine model was used to study the initiation of colonization by *Haemophilus influenzae* and *Streptococcus pneumoniae*. Both bacterial species, which occupy a similar microenvironment within the nasopharynx, persisted during colonization when given individually. Co-colonization, however, resulted in rapid clearance of *S. pneumoniae* from the upper respiratory tract, associated with increased recruitment of neutrophils into paranasal spaces. Systemic depletion of either neutrophil-like cells or complement was sufficient to eliminate this competitive effect, indicating that clearance was likely due to enhanced opsonophagocytic killing. The hypothesis that modulation of opsonophagocytic activity was responsible for host-mediated competition was tested using in vitro killing assays with elicited neutrophil-like cells. Components of *H. influenzae* (but not *S. pneumoniae*) stimulated complement-dependent phagocytic killing of *S. pneumoniae*. Thus, the recruitment and activation of neutrophils through selective microbial pattern recognition may underlie the *H. influenzae*–induced clearance of *S. pneumoniae*. This study demonstrates how innate immune responses may mediate competitive interactions between species and dictate the composition of the colonizing flora.

## Synopsis

Bacterial infection commonly begins with organisms that colonize and proliferate on mucosal surfaces. These microenvironments may be occupied by multiple microbial species, suggesting that successful colonizers are distinguished by their capacity to prevail over their competitors. This study examines interactions between two bacterial species that both colonize and infect the human upper respiratory tract. In a mouse model, strains of both *Haemophilus influenzae* and *Streptococcus pneumoniae* efficiently colonize the nasal mucosa when tested individually. In contrast, following co-inoculation, *H. influenzae* rapidly and completely outcompetes *S. pneumoniae*. This competitive effect is dependent on the local responses from the host in the form of a specific type of white blood cell (neutrophil) that acts to engulf and kill microorganisms that have been labeled by proteins that bind to microbial surfaces (complement). The results of this study show that recognition of microbial products from one species may activate inflammatory responses that promote the clearance of another competing species. This study also demonstrates how manipulations such as antibiotics or vaccines, which are meant to diminish the presence of a single pathogen, may inadvertently alter the competitive interactions of complex microbial communities.

## Introduction

For many microorganisms, including some that have the potential to behave as pathogens, their primary interaction with a host is through stable colonization of mucosal surfaces [1]. The composition of the flora that inhabits these sites is, in general, highly specific to a particular host species, suggesting that host factors must be an important determinant in the selection of colonizing microbes [1]. In few instances is the molecular basis of the host–microbial interaction that leads to this highly specific relationship understood.

Host–microbial relationships are commonly studied using experimental systems that examine single microbial species. Yet the mucosal surfaces where these organisms reside are often colonized with diverse populations comprised of different species. Successful occupants of such environments must have mechanisms that allow for their persistence by excluding potential competitors in a process referred to as microbial interference [2]. However, specific host or microbial mechanisms that promote or inhibit competitive interactions have not been characterized in vivo [3]. A more thorough understanding of such mechanisms is warranted, since the balance of competitive factors is increasingly being altered by the use of selective antimicrobials or vaccines that target a limited array of colonizing species or strains. In these situations, members of the microflora that might otherwise be suppressed may become predominant and, in some cases, the source of infection.

In prior studies, we have investigated competitive interactions between microbes using two distantly related prokaryotes: the gram-positive *Streptococcus pneumoniae* and the gram-negative *Haemophilus influenzae* [4–6]. Both species reside primarily on the mucosal surface of the human nasopharynx and under certain conditions are capable of causing a similar spectrum of disease [7,8]. *S. pneumoniae* and *H. influenzae* are among the most prevalent bacterial pathogens causing otitis media in children and community-acquired pneumonia or chronic bronchitis in adults [9–11]. Their shared niche may be a consequence of common mechanisms to promote colonization such as the expression of the cell-surface adhesin phosphorylcholine and a secreted protease that inactivates human immunoglobulin A1 [12–14]. The prevalence of asymptomatic carriage for both species may exceed 50% in some populations, especially infants [15,16]. This suggests that co-colonization might be common, and, therefore, that these species may have evolved specific mechanisms for targeting one another. Several such mechanisms, all of which predict that *S. pneumoniae* should prevail, have been studied in vitro. These include the rapid killing of *H. influenzae* by the toxic effects of hydrogen peroxide, which is generated at potentially bactericidal concentrations by the aerobic metabolism of *S. pneumoniae* [4]. In addition, a cell-surface neuraminidase expressed by *S. pneumoniae* is capable of removing sialic acid, a structure that decorates the surface of the lipopolysaccharide (LPS) of *H. influenzae* and contributes to its survival during infection [5].

The purpose of this study was to examine the interaction between these two species in vivo during experimental colonization. We demonstrate that these species compete in a murine model of carriage in a manner opposite to that predicted by in vitro investigation. Our findings demonstrate that species-specific stimulation of innate immune responses may be a determining factor in the outcome of polymicrobial interactions during colonization.

## Results

### Competition during Nasopharyngeal Colonization

**Figure 1:**
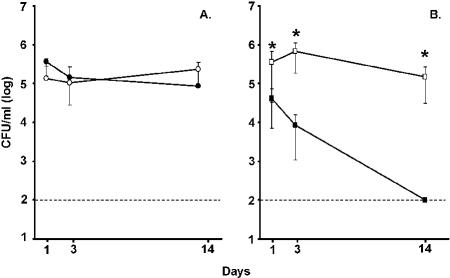
Colonization of BALB/c SCID Mice by *H. influenzae* Strain *Hi*636 and *S. pneumoniae* Strain *Sp*1121 The mean density of *H. influenzae* strain *Hi*636 (A) and *S. pneumoniae* strain *Sp*1121 (B) in upper respiratory tract lavage ± standard deviation was determined on the day indicated following intranasal inoculation with 1 × 10^7^ CFU of either one (open symbols) or both species (solid symbols). * *p <* 0.01 compared to co-colonization. Dashed line denotes the lower limit of detection.

**Figure 2:**
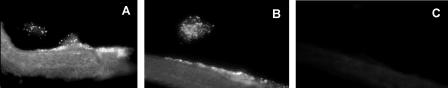
Immunofluorescence Showing Co-Localization of *H. influenzae* and *S. pneumoniae* in the Murine Nasopharynx BALB/c SCID mice were co-colonized with *Hi*636 and *Sp*1121, and at 24 h post-inoculation adjacent 5-μm frozen parasagittal tissue sections through the lateral nasal spaces of the same animals were stained with anti-capsular polysaccharide serum specific to type b *H. influenzae* (A), type 23F *S. pneumoniae* (B), or secondary antibody control with no primary antibody (C). Magnification, 400×.

In the course of establishing a mouse model for the colonization of the upper respiratory mucosa by *H. influenzae,* we found that BALB/c mice with severe combined immunodeficiency (SCID) were susceptible to chronic colonization of the upper respiratory tract with an encapsulated type b isolate (strain *Hi*636) (Figure 1A) [17]. Because SCID mice can also be colonized by encapsulated *S. pneumoniae* (strain *Sp*1121), this observation allowed for testing the effects of co-colonization (Figure 1B) [18]. Immunofluorescent staining of tissue sections from co-colonized mice showed that *Hi*636 and *Sp*1121 may co-localize in dense clusters along the epithelial surface and in mucoid material within the lumen of adjacent paranasal air spaces of the anterior nasopharynx (Figure 2). The presence of both species in the same microenvironment of co-colonized animals provided the rationale to analyze their competition either through direct bacterial–bacterial interaction or indirectly through the induction of local host responses.

Quantitative cultures of upper respiratory tract lavage fluid showed that over a 2-wk period there was stable colonization by *Hi*636 that was unaffected by co-colonization with *Sp*1121 (see Figure 1A). In contrast, colonization by *Sp*1121 was significantly reduced by day 1 post-inoculation in mice simultaneously challenged with *Hi*636 in the contralateral naris (*p <* 0.01; Figure 1B). By day 14 post-inoculation, there was no detectible *Sp*1121 in cultures obtained from dual inoculated animals. A similar competitive effect of *H. influenzae* on *S. pneumoniae* was observed even when *S. pneumoniae* colonization was pre-established by inoculation 24 h prior to intranasal challenge with *H. influenzae* (data not shown). Since the inhibition of *Hi*636 on *Sp*1121 colonization contrasted with the previously demonstrated bactericidal effect of *S. pneumoniae* on *H. influenzae* during co-culture in vitro, we pursued the hypothesis that a host response to the combined presence of both species mediates competition in vivo [4,19].

**Figure 3:**
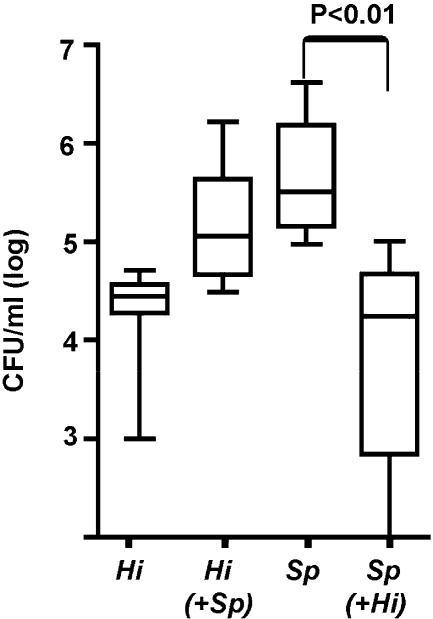
Competition between Species during Co-Colonization of Immunocompetent Mice The density of *H. influenzae* strain *Hi*636 *(Hi)* and *S. pneumoniae* strain *Sp*1121 *(Sp)* in upper respiratory tract lavage was determined at 24 h after intranasal inoculation of C3H/HeOuJ mice. Box-and-whiskers plot indicates high and low values, median, and interquartile ranges; *n* ≥ 10 in each group. Co-inoculated species shown in parentheses. The lower limit of detection for bacteria in lavage culture was 10^2^ CFU/ml.

The presence of competition in SCID animals indicated that the mechanism responsible for the inhibitory effect of *Hi*636 on *Sp*1121 colonization is independent of adaptive immunity. Subsequently, we demonstrated that immunocompetent C3H/HeOuJ mice were susceptible to nasopharyngeal colonization (>24 h at a density of >10^4^ CFU/ml of upper respiratory tract lavage) by *Hi*636 (Figure 3). In C3H/HeOuJ mice, a competitive effect of *Hi*636 on *Sp*1121 similar to that observed in SCID mice was also seen by 24 h post-inoculation (*p <* 0.01).

### Neutrophils and Complement Are Required for Competitive Interactions

**Figure 4:**
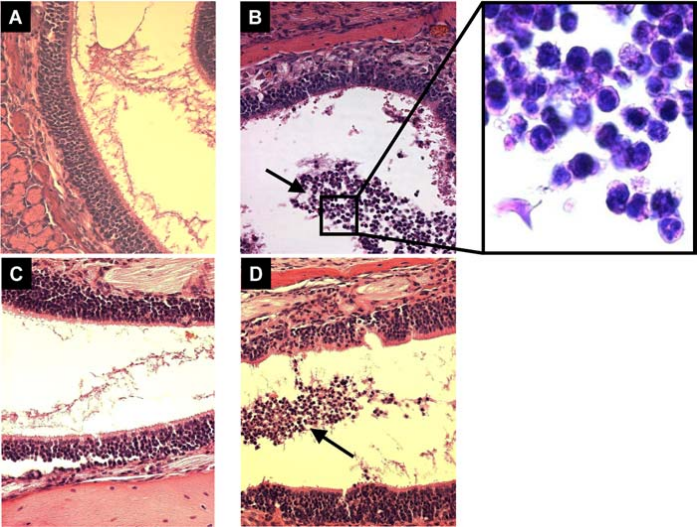
H&E-Stained Parasagittal Sections Showing the Luminal Space between Two Adjacent Nasal Turbinates Representative sections are shown for BALB/c SCID mice mock colonized (A) or co-colonized with *H. influenzae* strain *Hi*636 and *S. pneumoniae* strain *Sp*1121 (B); C3H/HeOuJ mice mock colonized (C) or co-colonized with *Hi*636 and *Sp*1121 (D). Arrows indicate cells infiltrating into the lumen of nasal spaces in co-colonized mice. Under higher magnification these cells have the morphologic appearance of neutrophils. Magnification, 400× (insert magnification, 1,000×).

**Figure 5:**
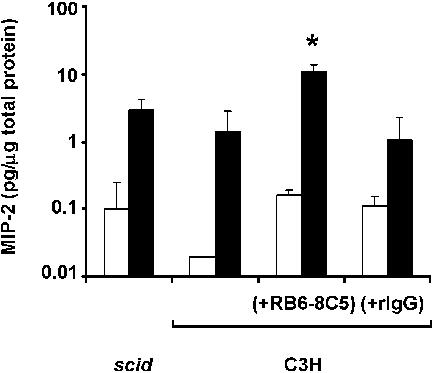
The Effect of Co-Colonization on the Concentration of MIP-2 in Upper Respiratory Tract Lavage Fluid MIP-2 levels normalized to total protein content were determined in mock colonized (open bars) and dual colonized (solid bars) mice including BALB/c SCID mice, C3H/HeOuJ mice, and C3H/HeOuJ mice treated with RB6-8C5 or rat IgG control as indicated. Values are geometric means ± standard deviation. *p < 0.01 compared to other co-colonized groups of C3H mice. Dual colonized SCID and C3H mice showed significantly higher levels of MIP-2 compared to mock colonized controls (*p <* 0.03).

Histopathological examination of nasopharyngeal sections showed a minimal cellular inflammatory response within the epithelium or subepithelium of mock or singly colonized SCID or immunocompetent mice (Figure 4A and 4C), as previously described [20]. Dual colonized animals, however, showed a marked influx of cells, with a predominance of neutrophils, confined to the lumen of the lateral nasal air spaces, indicative of an acute, localized, suppurative rhinitis (Figure 4B and 4D). The influx of these cells correlated with an increased concentration of macrophage inhibitory protein 2 (MIP-2), one of the murine CXC chemokines that can recruit neutrophils, in upper respiratory tract lavage fluid from co-colonized SCID and C3H mice (*p <* 0.03 compared to mock colonized; Figure 5).

**Figure 6:**
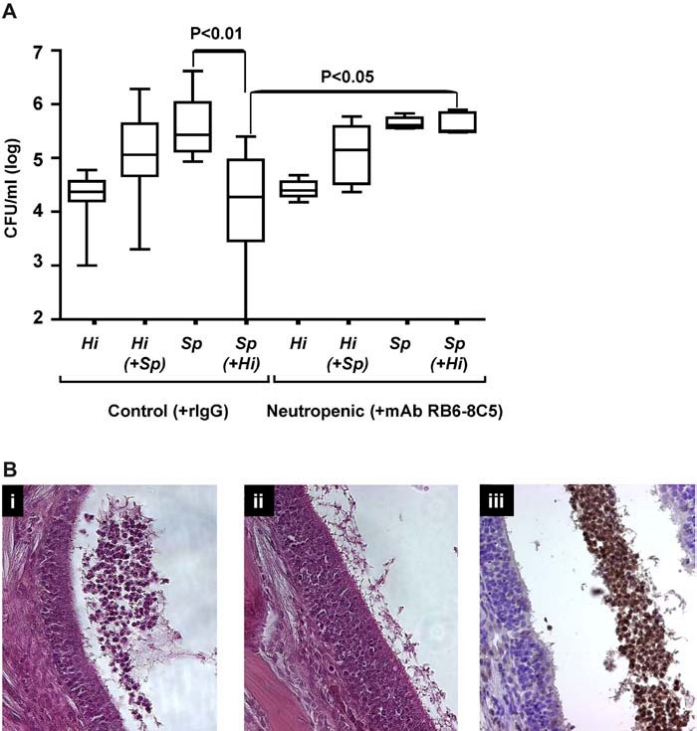
The Effect of Depletion of Neutrophil-Like Cells on Competition between Species during Co-Colonization (A) The density of *H. influenzae* strain *Hi*636 *(Hi)* and *S. pneumoniae* strain *Sp*1121 *(Sp)* in upper respiratory tract lavage was determined at 24 h after intranasal inoculation in C3H/HeOuJ mice pretreated with RB6-8C5 to deplete neutrophil-like cells or pretreated with rat IgG as a control. Box-and-whiskers plot indicates high and low values, median, and interquartile ranges; *n ≥* 5 in each group. Co-inoculated species shown in parentheses. The lower limit of detection for bacteria in lavage culture was 10^2^ CFU/ml. (B) Representative parasagittal sections of the lateral nasal tissues adjacent to the turbinates of co-colonized C3H/HeOuJ mice: (i) untreated control (H&E-stained), (ii) pretreated to deplete neutrophil-like cells (H&E-stained), or (iii) untreated control (stained with mAb Ly-6G to detect neutrophil-like cells). Magnification, 400×.

To examine whether the influx of neutrophils contributed to the competitive interaction between species, C3H/HeOuJ mice were pretreated with RB6-8C5, a rat monoclonal antibody (mAb) to murine Ly-6G expressed on neutrophil-like cells, prior to bacterial challenge. Treatment with RB6-8C5 but not control antibody resulted in complete loss of the inhibitory effect of *H. influenzae* on *S. pneumoniae* (Figure 6A). The effect of RB6-8C5 correlated with the depletion of neutrophils from peripheral blood and in the lumen of the lateral nasal spaces in tissue sections of co-colonized mice (Figure 6B). This absence of an influx of neutrophil-like cells in RB6-8C5-treated mice occurred despite significantly higher concentrations of MIP-2 compared to other co-colonized groups (*p <* 0.01) and was suggestive of a loss of feedback inhibition on expression of this chemokine (see Figure 5).

**Figure 7:**
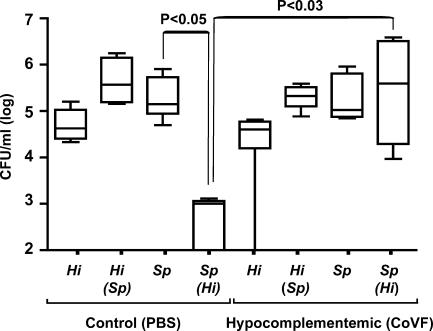
The Effect of Complement Depletion on Competition between Species during Co-Colonization The density of *H. influenzae* strain *Hi*636 *(Hi)* and *S. pneumoniae* strain *Sp*1121 *(Sp)* in upper respiratory tract lavage was determined at 24 h after intranasal inoculation in C3H/HeOuJ mice pretreated with cobra venom factor (CoVF) to deplete complement or PBS control. Box-and-whiskers plot indicates high and low values, median, and interquartile ranges; *n ≥* 5 in each group. Co-inoculated species shown in parentheses. The lower limit of detection for bacteria in lavage culture was 10^2^ CFU/ml.

A similar loss of the inhibitory effect of *H. influenzae* on *S. pneumoniae* colonization was seen in C3H/HeOuJ mice pretreated with cobra venom factor to deplete complement (Figure 7). Together these observations suggested that loss of pneumococci from the mucosal surface resulted from opsonization by complement, followed by phagocytic clearance by Ly-6G-positive neutrophil-like cells.

### Stimulation of Neutrophil-Mediated Killing by Innate Recognition of *H. influenzae*

**Figure 8:**
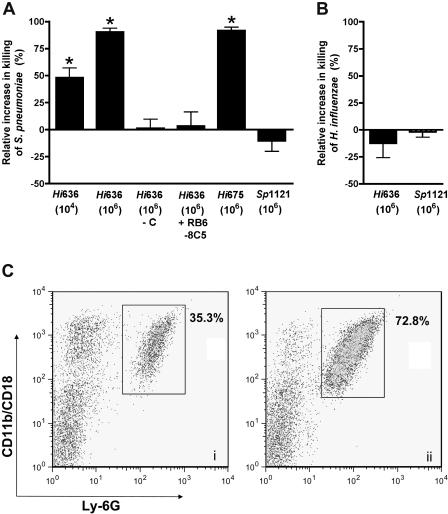
Stimulation of Neutrophil-Like Cells and Opsonophagocytic Killing by Components of *H. influenzae* Killing of *S. pneumoniae* strain *Sp*1121 (A) or *H. influenzae* strain *Hi*636 (B) by neutrophil-enriched PECs was determined over a 45-min incubation with active complement. The effect on killing by neutrophil-enriched PECs of i.p. administration of heat-inactivated whole bacteria at the dose indicated is shown relative to controls consisting of casein administration alone. No stimulation of killing was observed in controls using inactivated complement (−C) or cells from animals pretreated with mAb RB6-5C8. Values represent three or more independent determinations in duplicate ± standard error of the mean of the percent killing relative to controls using neutrophil-enriched PECs elicited without the addition of heat-inactivated bacteria. *p < 0.001 compared to groups without heat-inactivated *H. influenzae* and active complement. (C) A representative FACS analysis of neutrophil-enriched PECs showing the effect of i.p. administration of casein alone (i) or with 10^6^ heat-inactivated *Hi*636 (ii) on the proportion of Ly-6G positive cells co-expressing the activation marker CD11b/CD18 (boxed).

To test whether the inflammatory cells recruited in response to co-colonization were sufficient to account for interspecies competition, neutrophil-enriched peritoneal exudate cells (PECs) were analyzed in ex vivo killing assays using murine complement. Neutrophil-enriched PECs elicited by administration of heat-inactivated *Hi*636 (10^4^–10^6^ bacteria) in casein showed a dose-dependent increase in their ability to kill *S. pneumoniae* compared to controls consisting of casein alone (*p <* 0.001) (Figure 8A). *Hi*636 stimulation of neutrophil-enriched PECs, however, had no effect on survival of *H. influenzae* (Figure 8B). Increased pneumococcal killing by *H. influenzae–*stimulated neutrophil-enriched PECs required active complement (Figure 8A) but was independent of the presence of specific antibody, since serum obtained from SCID mice provided a sufficient source of complement for killing assays. Increased pneumococcal killing by *H. influenzae–*stimulated neutrophil-enriched PECs also correlated with an increase in the proportion of Ly-6G-positive PECs co-expressing the activation marker CD11b/CD18, which has recently been shown to be important in defense against pneumococcal infection (Figure 8C) [21]. Heat-inactivated *Hi*675, a non-typeable *H. influenzae* isolate, showed a similar capacity to elicit activated neutrophil-like cells and stimulate killing of *S. pneumoniae* (Figure 8A). In contrast to observations with heat-inactivated *H. influenzae,* intraperitoneal administration of equivalent doses of heat-inactivated *Sp*1121 neither stimulated activation of neutrophil-enriched PECs nor killing of either bacterial species (Figure 8A and 8B, and data not shown). Moreover, co-administration of heat-inactivated *Sp*1121 together with heat-inactivated *Hi*636 did not appear to add to levels of pneumococcal killing by neutrophil-enriched PECs compared to administration of heat-inactivated *Hi*636 alone (data not shown). Prior treatment of animals with RB6-8C5 mAb eliminated *H. influenzae–*induced killing of *S. pneumoniae* associated with the loss of elicited neutrophil-like cells, as also demonstrated during in vivo competition experiments (Figure 8A). Together these results suggest that the innate immune response to components of *H. influenzae* was sufficient to stimulate increased opsonophagocytic clearance of *S. pneumoniae* by neutrophil-like cells during co-colonization.

## Discussion

This study demonstrates that the composition of the colonizing flora may be affected by competition between multiple microbial species through the innate host responses they induce. Either of the two species analyzed in this report persisted on the mucosal surface of mice when given individually, but in combination one species quickly and consistently became predominant. This competitive relationship between species, moreover, was the result of the host's response to co-colonization and was not predicted by in vitro investigation of direct bacterial–bacterial interactions [4].

In the example of microbial interference described in this study, clearance of *S. pneumoniae* required both complement and the recruitment of neutrophil-like cells to the mucosal surface. A central role of these host factors was not unexpected since complement-mediated opsonization followed by ingestion and killing by professional phagocytes such as neutrophils or macrophages is a major host defense against this encapsulated gram-positive pathogen [22]. A further consideration in defining the contribution of these components of innate immunity is that inflammatory responses to polymicrobial colonization may be markedly different from those to a single type of organism. In an earlier report, we described the synergistic proinflammatory responses of respiratory epithelial cells in vitro and in the nasal mucosa in vivo when exposed to *H. influenzae* and *S. pneumoniae* in combination [20]. Discrete signals from each species contribute to levels of the neutrophil-recruiting chemokine MIP-2 (or IL-8 in human epithelial cells) significantly greater that seen with either organism alone. This synergistic response of the epithelium correlates with an influx of neutrophil-like cells into the nasal spaces. In the current study, which examines the outcome of these inflammatory responses, it was not practical to obtain adequate numbers of neutrophils directly from the nasal spaces to address whether these were sufficient for the clearance of *S. pneumoniae*. When tested ex vivo, murine neutrophils enriched from PECs did not kill *S. pneumoniae Sp*1121 efficiently. This suggested that the recruitment of neutrophils may not be sufficient to account for the competitive effect described here. Rather, stimulation of neutrophil-like cells with bacterial components of *H. influenzae* was required for efficient clearance of *S. pneumoniae*. Killing in these assays was dependent on active complement, consistent with its role as an opsonin promoting phagocytosis. If similar events occur in the local environment of the nasopharynx, innate immune responses, consisting of complement and enhanced neutrophil recruitment and activation through recognition of microbial products, may underlie the host's role in clearance of colonizing bacteria from the mucosal surface.

Activation and enhanced opsonophagocytosis of neutrophil-like cells ex vivo was found in response to products of *H. influenzae* but not equivalent doses of products from *S. pneumoniae*. The selective innate responses of inflammatory cells such as neutrophils to products from one microbe but not another, therefore, may provide a mechanism whereby one species induces clearance of a competitor. The LPS of *H. influenzae* and multiple other cellular components, including peptidoglycan, lipoproteins, phosphorylcholine, and an incompletely characterized soluble cytoplasmic fraction, have been implicated in promoting inflammatory responses [23–27]. Purified LPS from other species has been shown to trigger an increase in the migration, life span, and activity of neutrophils [28–30]. The molecular nature of the signal(s) from *H. influenzae* mediating the recruitment and activity of neutrophils is the topic of ongoing investigation. Results to date do not suggest that purified LPS of *H. influenzae* is sufficient to stimulate killing of *S. pneumoniae* by neutrophil-enriched PECs (data not shown). Although components of *S. pneumoniae,* including cell wall fragments and lipoteichoic acid and its toxin (pneumolysin), have been shown to be potent inducers of inflammation, these appeared to be at least 100-fold less active on a per cell basis in generating the neutrophil responses described here [31–34]. *H. influenzae* products, furthermore, stimulated killing of another species *(S. pneumoniae),* but had no effect in opsonophagocytic killing assays on the same species and strain from which these products were derived. Thus, our findings demonstrate that one species may compete with another through selective induction of host responses and may benefit from the differences in its susceptibility to the antimicrobial host factors it induces.

This study shows the importance of specific innate immune responses in dictating the initial success of a species in becoming established within a competitive niche such as the mucosal surface of the nasopharynx. Selective microbial pattern recognition, as demonstrated here for phagocytic activity, may act in the setting of a complex milieu of organisms that differ in their ability to trigger these host-specific responses. This process ultimately selects for the persistence of those species best able to evade the local host clearance factors induced by polymicrobial stimulaton of the innate immune system. The role of innate immunity in colonization described here is distinct from its more extensively studied role in infection.

An additional consideration is that the clearance of *S. pneumoniae* in co-colonized SCID mice demonstrates that the effects of complement and neutrophil-like cells were independent of adaptive immunity and the presence of antibody. Antibody-independent clearance was also demonstrated by in vitro assays in which we observed efficient killing in the presence of serum lacking anti-phagocytic antibodies. Antibody-independent opsonophagoctic killing of *S. pneumoniae,* as previously recognized in the classic studies of Wood et al., may be important in protection during the critical period prior to the acquisition of specific anti-capsular antibody [35]. Activation of phagocytic cells, however, has not been a feature of standardized opsonophagocytic killing assays for *S. pneumoniae* [36].

Colonization of mucosal surfaces is often the first step in the development of disease for many important pathogens. This study demonstrates that the presence of one species may impact the ability of another to persist in the same microenvironment on a mucosal surface. The focus of this report is on bacteria that commonly colonize and potentially infect the respiratory tract of humans. There may be clinical relevance to our observations that in a model of dual colonization *H. influenzae* was able to induce responses that caused the complete elimination of *S. pneumoniae,* a leading opportunistic pathogen. In regard to colonization, numerous surveys have described carriage rates for *H. influenzae* and *S. pneumoniae,* although, to our knowledge, none appear to have examined the effect of colonization by one species on the density of the other in a quantitative manner. In regard to disease involving the respiratory tract, however, some reports suggest that antagonism between these two species may occur in the natural host [37,38]. Most *H. influenzae* disease is currently caused by non-typeable strains, which were not tested in in vivo experiments, because of their less efficient colonization of either SCID or immunocompetent mice compared to the type b strain used in our study (data not shown). Nonetheless, an isolate of non-typeable *H. influenzae* was shown to be equally effective in stimulating neutrophil-mediated killing.

The competitive interactions described in this report may also be applicable to other combinations of microbes where there is evidence of antagonism in vivo. For example, a previously unrecognized competitive interaction between *S. pneumoniae* and *Staphylococcus aureus* could explain recent reports that children who receive the pneumococcal conjugate vaccine have lower rates of vaccine-type *S. pneumoniae* carriage, but higher rates of *Sta. aureus* nasal colonization as well as otitis media caused by *Sta. aureus* [39–41]. In this regard, the composition of the normal flora is generally regarded as a critical factor in protection from potentially more virulent opportunistic organisms. Our study provides an initial mechanistic understanding of how manipulation of the colonizing flora could have unexpected consequences on competitors. Since an expanding number of medical interventions impact the composition of the microflora, it would seem prudent to more fully appreciate the scope of competitive interactions on mucosal surfaces.

Our findings also demonstrate that the success of an organism in initiating carriage may depend on its ability to resist innate clearance mechanisms of mucosal surfaces generated in the setting of polymicrobial stimulation. Since characteristics that enhance evasion of innate immunity are often critical determinants of microbial pathogenicity, competition between species may promote the selection for virulence among species such as *S. pneumoniae* and *H. influenzae* that must first establish a niche on heavily colonized surfaces.

## Materials and Methods

### Bacterial strains and culture conditions.

*H. influenzae* and *S. pneumoniae* strains were grown as described elsewhere [42]. Strains used in vivo were selected because of their ability to efficiently colonize the murine nasopharynx and included *Hi*636 (a type b capsule-expressing, spontaneously streptomycin-resistant mutant of *H. influenzae* strain Eagan that was genetically modified to constitutively express phosphorylcholine), and *Sp*1121 (a type 23F capsule-expressing *S. pneumoniae* isolate from the human nasopharynx) [43,44]. *Hi*675 is a spontaneously streptomycin-resistant mutant of a non-typeable *H. influenzae* clinical isolate (A860516) provided from the collection of Dr. Loek van Alphen.

### Mouse model of nasopharyngeal colonization.

Six-week-old C.B-17/lcrCrlBR (BALB/c SCID, Charles River Laboratories, Wilmington, Massachusetts, United States) or C3H/HeOuJ (toll-like receptor 4 sufficient, Jackson Laboratory, Bar Harbor, Maine, United States) mice were housed in accordance with institutional animal care and use committee protocols. Mice were used in a previously described model of nasopharyngeal colonization with *S. pneumoniae* [18]. Briefly, groups of at least five mice per condition were inoculated intranasally with 10 μl containing 1 × 10^7^ CFU of PBS-washed, mid-log phase *H. influenzae,* *S. pneumoniae,* or both applied separately to each naris. Unless specified otherwise, 24 h post-inoculation the animal was sacrificed, the trachea cannulated, and 200 μl of PBS instilled. Lavage fluid was collected from the nares for determination of viable counts of bacteria in serial dilutions plated on selective medium containing antibiotics to inhibit the growth of contaminants (streptomycin, 100 μg/ml, to select for *H. influenzae* *Hi*636 and neomycin, 20 μg/ml, to select for *S. pneumoniae Sp*1121).

### Neutrophil and complement depletion.

mAb RB6-8C5, a rat anti-mouse IgG2b directed against Ly-6G on the surface of murine myeloid (and limited subpopulations of lymphoid) lineage cells , was purified from ascites of nude mice given the RB6-8C5 hybridoma [45,46]. To deplete neutrophils, 150 μg of mAb/animal was administered by intraperitoneal (i.p.) injection 24 h prior to intranasal challenge with bacteria. This dose was shown in pilot experiments to result in peripheral blood neutropenia (<50 granulocytes/μl) for a period of at least 48 h. Controls were given the equivalent i.p. dose of total rat IgG (Sigma, St. Louis, Missouri, United States).

Hypocomplementemia was induced by i.p. injection of 25 μg/animal of cobra venom factor (Quidel, San Diego, California, United States) in PBS 18 h prior to bacterial challenge. This procedure was previously shown to reduce levels of immunodetectible C3 to less than 3% of normal and result in a period of hypocomplementemia of greater than 48 h [47].

### Histology and immunofluorescence.

After the collection of lavage fluid, heads were fixed by serial overnight incubations in 4% paraformaldehyde, Decal (Decal Chemicals, Congers, New York, United States), and 70% ethanol. Paraffin-imbedded tissue was sectioned and stained with hematoxilin and eosin (H&E). For immunofluorescence, some samples were frozen using Tissue-Tek O.C.T. embedding medium (Sakura Finetek, Torrance, California, United States) in a Tissue-Tek Cryomold, and 5-μm-thick sections were cut, air dried, and fixed in acetone at 4 °C. Sections were rehydrated in PBS and incubated for 30 min in blocking solution of 5% normal goat serum in PBS. After washing in PBS, sections were incubated for 60 min at room temperature with primary antibody consisting of polyclonal rabbit anti–type b *H. influenzae* (DIFCO, Detroit, Michigan, United States) or anti-type 23F *S. pneumoniae* (Statens Serum Institut, Copenhagen, Denmark) diluted 1:1,000 in blocking solution. After further washing in PBS, secondary antibody (Texas Red–labeled goat anti-rabbit IgG, ICN Diagnostics, Orangeburg, New York, United States) was added in blocking solution for 60 min at room temperature and detected by fluorescence microscopy.

To label neutrophils, fixed, paraffin-embedded sections were rehydrated through a series of xylene and ethanol washes. Slides were then microwaved in 10 mM citric acid buffer (pH 6.0) and quenched for endogonous peroxidases with 3% hydrogen peroxide. Endogenous biotin was blocked with an Avidin-Biotin blocking kit (Vector Laboratories, Burlingame, California, United States) followed by a peptide blocking reagent (Coulter Immunotech, Hialeah, Florida, United States). Anti-mouse Ly-6G primary antibody was diluted to 1:1,000 in PBS containing 0.1% BSA and 0.2% Triton X-100 for overnight incubation at 4 °C. A biotinylated anti-rat secondary antibody was added for 30 min at 37 °C followed by avidin-horseradish peroxidase ABC reagent (Vector Laboratories). Signal was developed using DAB kit (Vector Laboratories).

### Measurement of MIP-2 concentration.

Upper respiratory tract lavage fluid was assayed for the concentration of MIP-2 by ELISA in duplicate according to the manufacturer's instructions (PharMingen, San Diego, California, United States). These values were normalized to the total protein of these samples (micro BCA protein assay, Pierce Biotechnology, Rockford, Illinois, United States).

### Isolation and characterization of murine neutrophils.

Neutrophil-enriched PECs were isolated from C3H/HeOuJ mice as previously described [48]. Briefly, phagocytes were obtained by lavage of the peritoneal cavity (8 ml/animal with PBS containing 0.02 M EDTA) of mice treated 24 h and again 2 h prior to cell harvest by i.p. administration of 10% casein in PBS (1 ml per dose). Cells collected from the peritoneal cavity cells were enriched for neutrophils using separation in a Ficoll density gradient centrifugation using Mono-Poly Resolving Medium according to the manufacturer's instructions (MP Biomedicals, Irvine, California, United States). This neutrophil-enriched fraction was collected and washed with 5 ml of Hank's buffer without Ca^++^ or Mg^++^ (Invitrogen, Carlsbad, California, United States) plus 0.1% gelatin. An aliquot of these cells was characterized using FACS for staining of granulocytes with anti-mouse Ly-6G mAb (BD Biosciences, San Jose, California, United States) [49]. Expression of the cell-surface activation marker CD11b/CD18 (BD Biosciences) was quantified for cells co-staining for expression of Ly-6G [50]. Where indicated, heat-inactivated *H. influenzae* (*Hi*636 or *Hi*675) and *S. pneumoniae* (*Sp*1121) were co-administered with the casein solution. PBS-washed, mid-log phase bacteria were heat-inactivated by treatment at 65 °C for 30 min and shown to be non-viable.

### Phagocytic killing assays.

Neutrophil-enriched PECs were counted by trypan blue staining and adjusted to a density of 7 × 10^6^ cells/ml. Killing during a 45-min incubation at 37 °C with rotation was assessed by combining 10^2^ PBS-washed, mid-log phase bacteria (in 10 μl) with complement source (in 20 μl), 10^5^ mouse phagocytes (in 40 μl), and Hank's buffer with Ca^++^ and Mg^++^ (Gibco, San Diego, California, United States) plus 1% gelatin (130 μl). Earlier time points and fewer effector cells relative to the number of target cells were shown in pilot experiments to result in less killing. The complement source consisted of serum from either SCID or X-linked immunodeficient mice (CBA/CaHN-Btkxid/J) previously shown to lack opsonophagocytic antibody to thymus-independent type 2 antigens including pneumococcal capsular polysaccharide and phosphorylcholine [51]. After stopping the reaction by incubation at 4 °C, viable counts were determined in serial dilutions. Percent killing was determined relative to the same experimental condition without i.p. administration of bacterial products (casein alone). No loss of bacterial viability was seen in controls using heat-inactivated complement (56 °C for 30 min). Additional controls consisting of heat-inactivated *Hi*636 administered without casein gave similar levels of killing, confirming that killing was stimulated by bacterial products rather than by casein.

### Statistical analysis.

Statistical comparisons of colonization among groups were made by the Kruskal-Wallis test with Dunn's post-test (Prism 4, GraphPad Software, San Diego, California, United States). In vitro killing assays were compared by ANOVA.

## Abbreviations

H&E: hematoxylin and eosin
i.p.: intraperitoneal
LPS: lipopolysaccharide
mAb: monoclonal antibody
MIP-2: macrophage inhibitory protein 2
PEC: peritoneal exudate cell
SCID: severe combined immunodeficiency
